# Identifying DNA methylation types and methylated base positions from bacteria using nanopore sequencing with multi-scale neural network

**DOI:** 10.1093/bioinformatics/btaf397

**Published:** 2025-07-14

**Authors:** Zheng Chen, Peng Ni, Jianxin Wang

**Affiliations:** School of Computer Science and Engineering, Central South University, Changsha 410083, China; Xiangjiang Laboratory, Changsha 410205, China; Hunan Provincial Key Lab on Bioinformatics, Central South University, Changsha 410083, China; School of Computer Science and Engineering, Central South University, Changsha 410083, China; Xiangjiang Laboratory, Changsha 410205, China; Hunan Provincial Key Lab on Bioinformatics, Central South University, Changsha 410083, China; School of Computer Science and Engineering, Central South University, Changsha 410083, China; Xiangjiang Laboratory, Changsha 410205, China; Hunan Provincial Key Lab on Bioinformatics, Central South University, Changsha 410083, China

## Abstract

**Motivation:**

DNA methylation plays important roles in various cellular physiological processes in bacteria. Nanopore sequencing has shown the ability to identify different types of DNA methylation from individual bacteria directly. However, existing methods for identifying bacterial methylomes showed inconsistent performances in different methylation motifs in bacteria and didn’t fully utilize the different scale information contained in nanopore signals.

**Results:**

We propose a deep-learning method, called Nanoident, for de novo detection of DNA methylation types and methylated base positions in bacteria using Nanopore sequencing. For each targeted motif sequence, Nanoident utilizes five different features, including statistical features extracted from both the nanopore raw signals and the basecalling results of the motif. All the five features are processed by a multi-scale neural network in Nanoident, which extracts information from different receptive fields of the features. The Leave-One-Out Cross-Validation (LOOCV) on the dataset containing 7 bacteria samples with 46 methylation motifs shows that Nanoident achieves ∼10% improvement in accuracy than the previous method. Furthermore, Nanoident achieves ∼13% improvement in accuracy in an independent dataset, which contains 12 methylation motifs. Additionally, we optimize the pipeline for de novo methylation motif enrichment, enabling the discovery of novel methylation motifs.

**Availability and implementation:**

The source code of Nanoident is freely available at https://github.com/cz-csu/Nanoident and https://doi.org/10.6084/m9.figshare.29252264.

## 1 Introduction

There are primarily three types of methylation found in bacteria: N4-methylcytosine (4mC), 5-methylcytosine (5mC), and N6-methyladenine (6 mA) (Sánchez-Romero *et al.* 2015). These methylation types play crucial roles in various bacterial processes, such as maintaining genome integrity and regulating gene expression (Gonzalez *et al.* 2014).

Studies have revealed that DNA methylation in bacteria is highly motif-driven. When a motif is targeted by methyltransferases (MTases), typically more than 95% of the sequences are methylated (Beaulaurier *et al.* 2019). DNA motifs are defined as nucleic acid sequences with specific biological significance and may contain distinct binding sites (Das and Dai 2007). Therefore, identifying methylation motifs is beneficial for studying bacterial physiological functions and epigenetic modifications (Wang *et al.* 2019). Traditionally, binding motifs have been determined through DNase footprinting, gel-shift assays, or reporter construct assays. Nowadays, more efficient computational methods have been developed, such as MEME (Bailey and Elkan 1994). These methods discover motif sequences by searching for overrepresented DNA patterns and associating them with previously characterized sets from the literature and public databases (Blatti and Sinha 2014).

After detecting the motif sequence, the next step is to identify the corresponding methylation type and the methylated base position within the motif. The identified motifs can then be utilized as auxiliary input for software such as ModKit (https://github.com/nanoporetech/modkit), which aids in further generating genome-wide methylation profiles. Additionally, we can obtain the corresponding restriction-modification (R-M) system of the identified motifs by searching the methyltransferase database (Beaulaurier *et al.* 2019). We can also use downstream tools like MicrobeMod (Crits-Christoph *et al.* 2023) to verify the functionality of the corresponding R-M genes, confirm their activity, and enhance the accuracy of annotations. Nahar *et al.* utilized identified motifs to study type I and type III R-M systems of *Actinobacillus pleuropneumoniae* (Nahar *et al.* 2023), which regulate different phase variables through phase-variable expression, thus affecting key bacterial characteristics such as virulence, immune evasion, and antibiotic resistance. This research contributes to a better understanding of bacterial regulatory mechanisms and informs the development of vaccines against these pathogens. Furthermore, the identified motifs can be used as features for metagenome methylation binning (Tourancheau *et al.* 2021).

The determination of motifs through biochemical experiments is a time-consuming process. NT-seq (Li *et al.* 2022) can effectively map multiple types of methylation, but it requires complex chemical treatments to obtain a genome-wide methylation spectrum. Thus, it is necessary for biochemical researchers to develop efficient and user-friendly computational methods. Nanopore sequencing has proven to be a promising method for detecting DNA modifications (Laszlo *et al.* 2013). However, most existing methods can detect only a single type of methylation (e.g. Nanopolish; Simpson *et al.* 2017), require training a separate model for each methylation type (e.g. PoreFormer; Dai *et al.* 2024), or focus solely on the modification of a single site without exploring the shared properties within the same motif (e.g. Tombo [Stoiber *et al.* 2016], mCaller [McIntyre *et al.* 2019]). None of these methods have been applied to identify unknown bacterial methylomes without relying on prior knowledge. Following the rationale that nearly every occurrence (>95%) of a methylation sequence motif in bacteria is methylated, Tourancheau *et al.* developed a multi-label classification framework, called nanodisco (Tourancheau *et al.* 2021), using multiple machine-learning classifiers (such as k-nearest neighbors, random forest, and neural networks) for methylation motif typing (the identification of methylation types) and fine mapping (the identification of methylated base positions). However, nanodisco is not accurate enough.

It has been demonstrated that in nanopore sequencing, the signal differences between native (NAT) sequencing and whole-genome amplification (WGA) sequencing diminish as the distance from the methylation site increases (Tourancheau *et al.* 2021). On the one hand, extracting features on a smaller scale is more reliable because it is closer to the affected methylation sites, and on the other hand, large-scale feature extraction contains more data points and provides richer information. Therefore, it is crucial to integrate multi-scale information effectively. Additionally, experiments have shown that DNA methylation introduces differences in basecalling errors (Bonet *et al.* 2022) and pore passage times in nanopore sequencing (Yu *et al.* 2017). However, these features have not yet been comprehensively exploited. In this study, we introduce a novel method called Nanoident, which integrates nanopore signals and basecalling features with a multi-scale neural network. Nanoident mitigates the adverse effects of data noise by leveraging multi-source features and extracting multi-scale information through convolutional kernels of varying sizes. Our results show that, in comparison to existing methods, Nanoident significantly enhances performance in de novo methylation typing and fine mapping in bacteria. Furthermore, we have optimized the de novo motif enrichment module in the nanodisco pipeline, enabling it to identify more motifs that were previously undetectable in nanodisco’s automatic mode.

## 2 Materials and methods

### 2.1 Nanopore sequencing data and the reference genomes

All data used in this work are available at the Sequence Read Archive under the BioProject PRJNA559199 (Tourancheau *et al.* 2021). Seven species of bacteria were used for training: *Bacillus amyloliquefaciens* H, *Bacillus fusiformis* 1226, *Clostridium perfringens* ATCC 13124, *Escherichia coli* K-12 substr. MG1655 ATCC 47076, *Helicobacter pylori* JP26, *Methanospirillum hungatei* JF-1, *Neisseria gonorrhoeae* FA 1090, *Nocardia otitidiscaviarum* NEB252, and *Thermacetogenium phaeum* DSM 12270. There are 46 different motifs in these seven bacteria, including 7 4mC motifs, 11 5mC motifs, and 28 6 mA motifs. The 46 motifs appear a total of 308 773 times across the genomes of all bacteria. The frequency of occurrence of different motifs also varies significantly. GT6mAC appeared the least (198 times), while G6mATC appeared the most frequently (44 388 times). An independent dataset containing nanopore reads from two bacteria (*N. otitidiscaviarum* NEB252 and *T. phaeum* DSM 12270) was used for testing. There are 12 different motifs in these two species, including 6 4mC motifs, 1 5mC motif, and 5 6 mA motifs. The 12 motifs appear a total of 102 055 times across the two bacteria. Each bacterium contains two types of samples (WGA samples and NAT samples). The reference sequences for the bacterial samples are available at NCBI with the following accession codes: CP041693, CP041696, NC_008261.1, CP014225.1, CP023448.1, NC_007796.1, NC_002946.2, CP041695, and CP003732. Methylation motifs of these bacteria were collected from the REBASE database. More detailed information about the data is provided in Tables 1 and 2, available as supplementary data at *Bioinformatics* online.

### 2.2 Our methods

#### 2.2.1 Data preprocessing

Nanopore sequencing reads were basecalled first. The reads from the forward and reverse strands in the raw files were stored separately. BWA-MEM (Li 2013) (v.0.7.15) and Rsamtools (Morgan *et al.* 2016) (v.1.34.1) were used to index, sort, and align the raw reads. Subsequently, using Nanopolish (Loman *et al.* 2015) (v.0.11.0), nanopore signals were re-squiggled to the reference genome. Finally, we calculated the difference in mean signal values at each site between the NAT sample and the WGA sample. We also processed the mean standard deviation difference and the mean dwell time difference. Additionally, we calculated the Mann–Whitney U-test *P*-value for the corresponding sites between the two samples.

**Figure 1.**
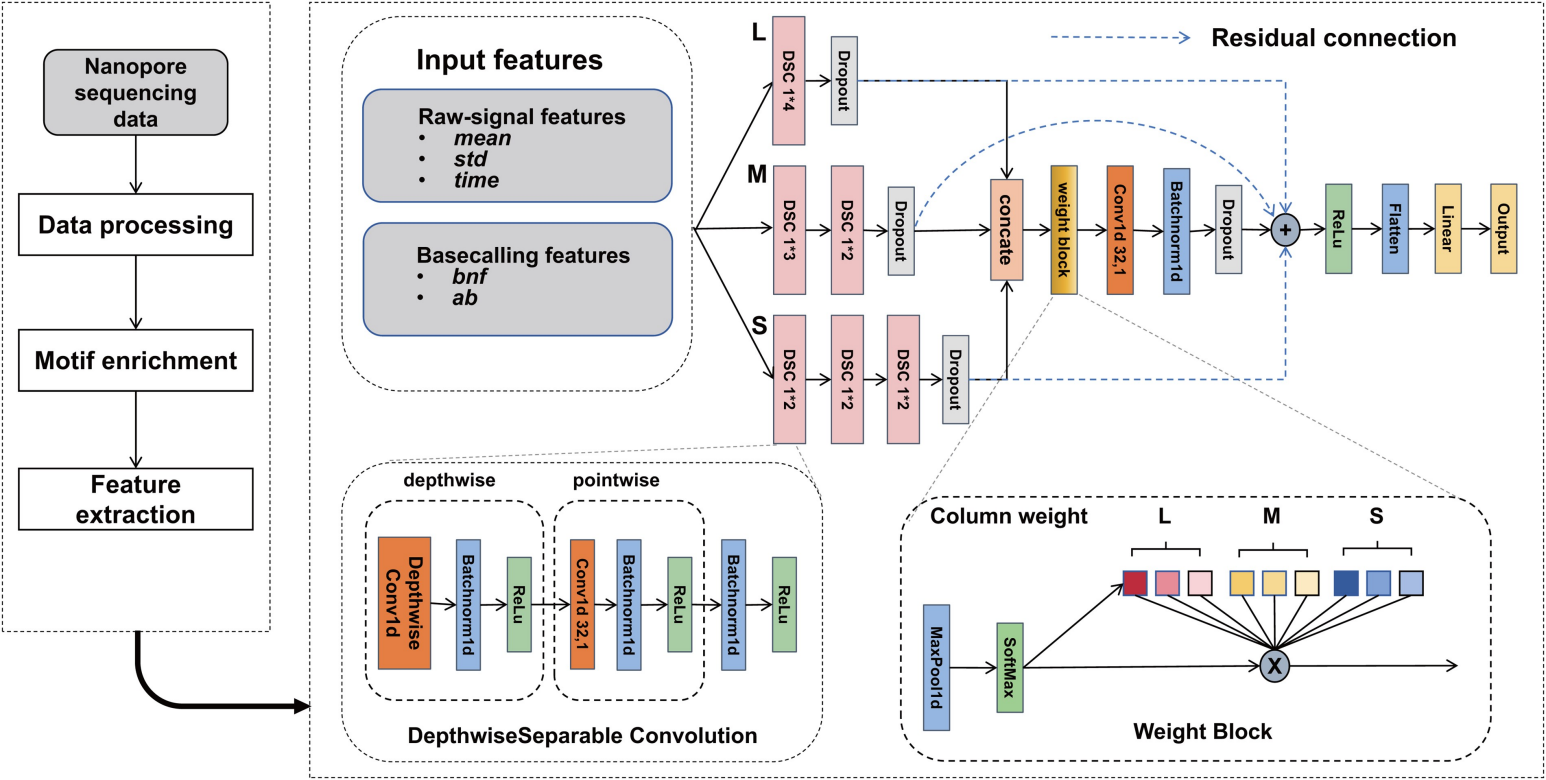
The pipeline and model architecture of Nanoident (n*m (e.g. 1*4) in DSC represents the convolution kernel size).

The pipeline for data preprocessing is illustrated in Fig. 1A, available as supplementary data at *Bioinformatics* online. After filtering out outliers from the nanopore data using Tukey’s fences method, we utilized bam-readcount (Khanna *et al.* 2021) to generate the proportion of matched bases in reads at each site and the quality score of basecalling-matched bases in reads.

#### 2.2.2 Feature extraction

**Figure 2.**
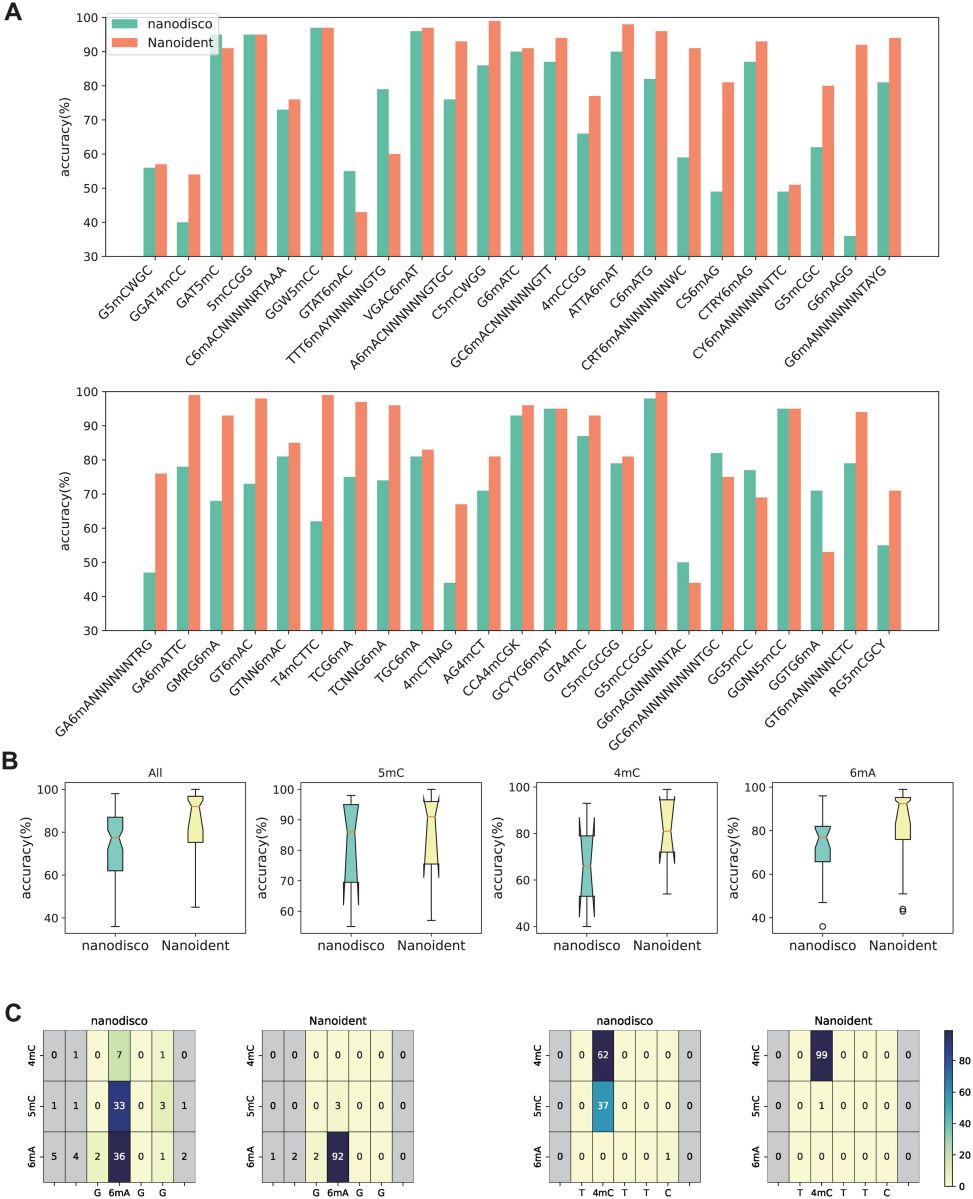
LOOCV evaluation. (A) Methylation typing and fine mapping accuracy of 46 motifs. (B) Two examples of probabilistic heat maps predicted by nanodisco and Nanoident. (C) Box plots of methylation typing and fine mapping accuracy for 46 motifs, divided by different methylation types.

After data preprocessing, we extracted five types of features from the nanopore data, including three statistical characteristics of nanopore signals and two basecalling features. The three signal features are: the mean difference (*mean*), the standard deviation difference (*std*), and the dwell time difference (*time*) in signal values. The two basecalling features are: the average quality score difference (*ab*) and the proportion of reference base differences processed by readcount (Khanna *et al.* 2021) (base_num_fraction, *bnf*). As shown in Fig. 2, available as supplementary data at *Bioinformatics* online, all five features exhibit distinct signatures across the three types of methylation. We also processed the corresponding base of each site for position correction in the postprocessing step.

For the training data, feature extraction was performed as follows:

1. For each targeted motif, we searched for all related locations of the motif in the reference genome using string matching. We then extracted the corresponding segments of nanopore signal data from all identified motif locations.
2. For each segment of nanopore signal data, we extracted features at two distinct lengths:
  1. We extracted nanopore signals within the range of [−14, 7] surrounding the methylation site to exclude any signals that contain overlapping regions. The purpose of this extraction is to eliminate the situation where the methylation site of one data sample falls within the feature range extracted from other samples.
  2. We extracted nanopore signals within the range of [−9, 2] surrounding the methylation site and labeled this as the 0 offset. Next, we extracted the range of [−8, 3] and labeled it as the 1 offset. This process continued, applying left and right offsets a total of seven times. Each feature was also labeled with the corresponding methylation type. Finally, the features were categorized into 21 distinct classes in the form of binary groups (methylation, offset), which included three types of methylation (4mC, 5mC, 6mA) and seven offset sites within the interval of [−3, 3].

Fig. 1B, available as supplementary data at *Bioinformatics* online illustrates the feature extraction process. We first utilize the de novo motif enrichment module to detect new motif sequences and extract their features. The feature extraction for model inference differs slightly from that of model training. Initially, the relevant locations of the desired inference motif within the DNA sequence are identified through string matching. Subsequently, the corresponding intervals encompassing the 12 sites preceding the start of the motif and the 5 sites following its end are extracted to calculate the associated *P*-value for the region, utilizing a sliding average with a window size of 5. The site exhibiting the maximum *P*-value is designated as the predicted methylation site. The subsequent procedure is similar to the training process, where the methylation site serves as the origin for feature extraction. However, only the signals from the interval [−9, 2] are extracted for input into the model for prediction.

#### 2.2.3 Model architecture of Nanoident

The pipeline and model structure of Nanoident are depicted in Fig. 1. In Nanoident, we employ Depthwise Separable Convolution (DSC) for feature processing. DSC comprises two components: depthwise convolution and pointwise convolution, which are utilized to extract spatial and channel features, respectively.

In Nanoident, we use one-dimensional convolution to capture spatial information from the one-dimensional nanopore signal input. We established three convolutional columns with varying receptive fields, designated as large (L), medium (M), and small (S), referred to as the Multi-Scale Convolutional module. This module is designed to extract different scales of information from features. Each column comprises one or more DSC blocks, and the outputs from the three columns are concatenated at a uniform scale. The large-column convolution consists of a single DSC block with a convolution kernel size of 4, the medium-column convolution consists of two DSC blocks with convolution kernel sizes of 3 and 2, and the small-column convolution consists of three DSC blocks, each using a convolution kernel size of 2.

In the feature extraction step, we processed five features (*mean*, *std*, *time*, *bnf*, and *ab*), each represented as a 12-dimensional vector. Initially, these five features are combined and subsequently input into the Multi-Scale Convolutional module. The outputs from the L, M, and S columns are then concatenated and subjected to a weight block, which assigns different weights to the different column-scale features, thereby focusing more attention on the more important features. After weighting, we use a pointwise convolution to integrate information across different scales. We implement residual connections between the pre-fusion and post-fusion convolution results to alleviate gradient vanishing, gradient explosion, and degradation of the network in deep architectures. Finally, we flatten the output and apply a linear layer for classification.

#### 2.2.4 Model training and evaluation

The training dataset comprises seven bacterial species. Prior to the training process, we implement data balancing by randomly downsampling each class to the minimum size. Subsequently, the dataset is randomly partitioned into a training set and a validation set in a 95:5 ratio. We establish a batch size of 256 and employ the Adam optimizer with default parameters. Training is terminated when the validation set loss decreases by less than 1e-3 over five consecutive epochs. We adopt Focal Loss during the training of Nanoident. Focal Loss is a loss function commonly used to allocate increased weight to samples that are more challenging to classify.

We splice different features of the same position, input them into the Nanoident model for training and inference, and then go through a postprocesing module to correct the position (illustrated in Fig. 1C, available as supplementary data at *Bioinformatics* online). In the position correction module, we first process the base sequence of the corresponding position segment and judge whether the methylation type output by the model is consistent with the base at the predicted site. If not, we continue to evaluate the site with the second highest prediction probability of the model and keep iterating until a matching site is found. (e.g. if the model predicts 5mC, the base at the predicted location needs to be C.)

We performed Leave-One-Out Cross-Validation (LOOCV) evaluation as nanodisco did by selecting data from 45 motifs out of 46 for training while evaluating on the remaining motif. We did not enable post-processing at this part in order to fairly compare the model performance improvements of Nanoident over nanodisco. Furthermore, we conducted training on all 46 motifs and evaluated the model’s performance on an independent dataset comprising two bacterial species.

## 3 Results

### 3.1 LOOCV evaluation

We first utilized the nanopore sequencing data of seven bacteria to evaluate the de novo methylation typing and fine mapping accuracy of Nanoident. To evaluate generalization of Nanoident, we used LOOCV evaluation, training on 45 motifs and validating on 1 left. We also retrained nanodisco using the mean feature of the processed data and found that it was consistent with the results mentioned in nanodisco paper (Table 3, available as supplementary data at *Bioinformatics* online). The comparison results of Nanoident and nanodisco are presented in Fig. 2A and Table 4, available as supplementary data at *Bioinformatics* online. Results show that the accuracies of Nanoident surpassed those of nanodisco in 35 out of 46 motifs. The average accuracy of Nanoident was 83.68%, which is 10% higher than that of nanodisco (73.93%). *H. pylori* JP26 showed the most improvement, with average accuracy increasing by nearly 20%. On 21 out of 46 motifs, Nanoident achieved >10% improvement in classification accuracy compared to nanodisco. For example, on the G6mACC motif, nanodisco achieved the lowest classification accuracy (36%), while Nanoident achieved 92% accuracy.

Fig. 2B compares predicted probabilities for G6mAGG and T4mCTTC. Nanodisco identifies methylation sites well but struggles with type classification. For instance, it assigned G6mAGG to 5mC with a probability of 33%, while only 36% probability was assigned to the correct methylation type, 6 mA. A similar pattern was observed for the T4mCTTC motif, where nanodisco predicted it as 5mC with a probability of 37%. In contrast, Nanoident accurately predicted the methylation types of G6mAGG and T4mCTTC with probabilities of 99% and 100%, respectively, and achieved de novo typing and fine mapping of the two motifs with accuracies of 92% and 99%, respectively.

Fig. 2C shows the summarized accuracies of Nanoident and nanodisco across 46 motifs. The results indicate that the overall performance of Nanoident is significantly superior to nanodisco. The variability of the accuracies across different motifs is notably reduced, indicating that Nanoident is more stable and generalized. The average accuracies of Nanoident for the 5mC, 4mC, and 6 mA motifs improved from 81.36%, 66.14%, 72.96% to 85.00%, and 81.00%, 83.50%, respectively. Although nanopore sequencing data is not suitable for the detection of 4mC and 6 mA, the detection accuracy is greatly improved under our method.

**Figure 3.**
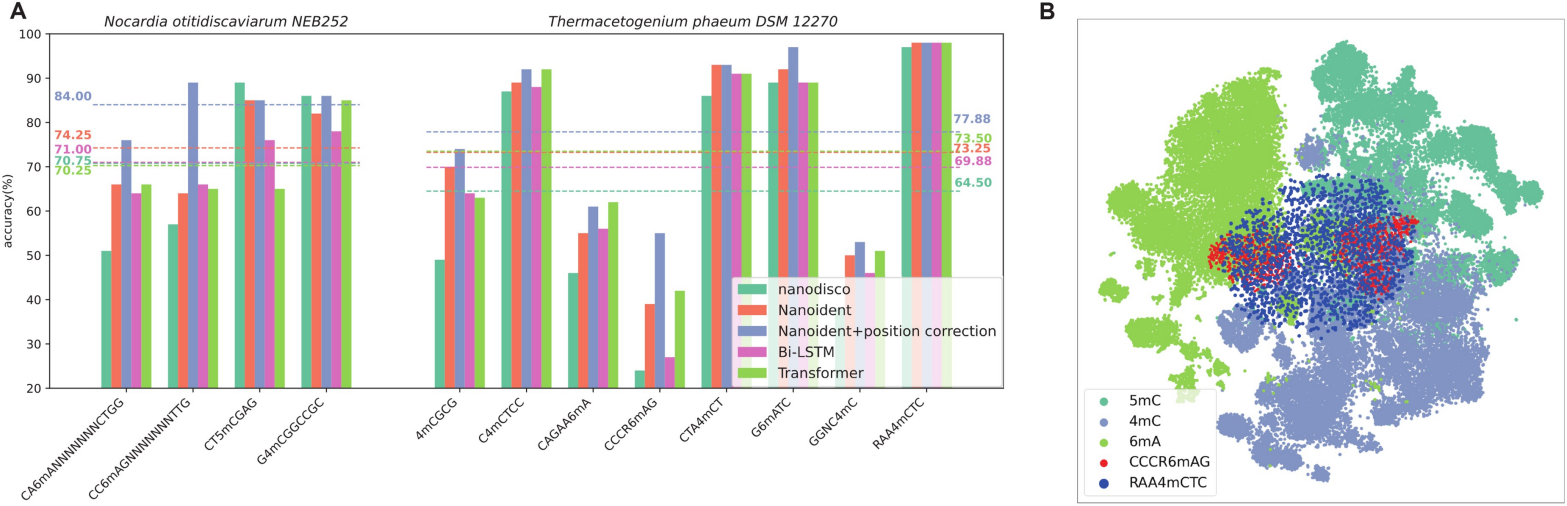
Evaluation on an independent dataset. (A) Comparison of accuracies among different methods on two independent bacterial datasets (the dashed line indicates the average accuracy of each method). (B) Mean feature distribution of 46 motifs (contained 4mC, 5mC, and 6 mA) in the training data, CCCR6mAG motif, and RAA4mCTC motif in the testing data after t-SNE reduction.

Nanodisco employs some older machine learning methods. However, nowadays, several classic deep learning methods have been proven to perform well in many tasks, such as Bi-directional Long Short-Term Memory (Bi-LSTM) and Transformer. Many top-performing methylation models adopt these structures, including DeepMod2 (Ahsan *et al.* 2024) and Rockfish (Stanojević *et al.* 2024). Therefore, in addition to the convolutional neural network used by Nanoident, we also attempted these two additional deep learning methods (Fig. 3, available as supplementary data at *Bioinformatics* online). In the LOOCV evaluation, Bi-LSTM and Transformer achieved average accuracies of 71.51% and 67.61%, respectively, both of which were lower than nanodisco. We believe the possible reason lies in the excessive number of linear layers in these two models. In our experiments, when models have too many linear layers, their accuracy is generally not high. We think this is because the scale of our input data is relatively small, and an excessive number of linear layers significantly increases the complexity of the model, thereby increasing the risk of overfitting. The parameter count of Nanoident is 14 319, while that of a two-layer Bi-LSTM and a one-layer Transformer is 36 693 and 27 733, respectively, far exceeding that of Nanoident. This is why we did not consider using more complex LSTM and Transformer models. The results of these two models are shown in Table 5, available as supplementary data at *Bioinformatics* online.

### 3.2 Evaluation of Nanoident on an independent dataset

We then evaluated Nanoident using an independent dataset comprising two bacterial species, *N. otitidiscaviarum* NEB252 and *T. phaeum* DSM 12270, which contained a total of 12 motifs. The results are presented in Fig. 3A and Table 6, available as supplementary data at *Bioinformatics* online. Nanoident improved accuracy for 10 of 12 motifs, with increases of ∼3.5% in *N. otitidiscaviarum* NEB252 and ∼9% in *T. phaeum* DSM 12270. In comparison with the nanodisco, Nanoident increased the overall average accuracy of the independent test set from 66.58% to 73.58%, reflecting an improvement of 7%. The Bi-LSTM and Transformer models achieved average accuracies of 70.25% and 72.42%, respectively, demonstrating that these two deep learning models still have certain advantages for some bacterial data. The independent dataset confirms Nanoident’s robustness in methylation typing and fine mapping. Subsequently, we added a position correction post-processing module, which further increased accuracy to 79.92% (+6.34%). We also added the post-processing module to nanodisco, which increased the average accuracy by 7.2%, demonstrating the effectiveness of the post-processing module.

We observed that for some motifs (e.g. CCCR6mAG), all models exhibited poor accuracies, while for others (e.g. RAA4mCTC), all models achieved high accuracies. We plotted the distribution of the mean features of the 46 motifs in training data and these two motifs after dimensionality reduction using t-SNE (Van der Maaten and Hinton 2008) (Fig. 3B). CCCR6mAG split into two red clusters, overlapping with both 6 mA and 4mC/5mC regions. It was inconsistent with the distribution of training data, making it difficult for the model trained solely on the existing data to adapt to the CCCR6mAG motif. This may suggest the need to expand the dataset for training so that the model can learn the common rules of different bacterial species and motifs. For RAA4mCTC, the cluster mainly fell in the 4mC region of the training data, and the rules learned from the training data were applicable to this motif, allowing different models to achieve better results.

### 3.3 The interpretability of Nanoident

To elucidate the effectiveness of the Multi-Scale Neural Network used in Nanoident, we evaluated each of the three convolutional networks at different scales using LOOCV evaluation (Table 7, available as supplementary data at *Bioinformatics* online). Our results indicate that multi-scale fusion networks achieve higher accuracy (83.68%) compared to single-scale networks (S scale: 83.55%, M scale: 82.17%, L scale: 78.56%).

Additionally, we evaluated each of the three convolutional networks at different scales (Table 8, available as supplementary data at *Bioinformatics* online) on five features separately (Table 9, available as supplementary data at *Bioinformatics* online) using an independent dataset. Independent tests confirmed multi-scale superiority (73.58% vs. S: 72.5%, M: 71.67%, L: 70.58%). The results also show that each feature also gets different classification accuracies with different scales of convolutional networks. For example, the mean feature achieves 71% accuracy with the S and L networks, which is 2% higher than with the M network. The *ab* and *bnf* features achieve the highest accuracy with the S network. Notably, the *ab* feature shows significantly different performance with the S and L networks. In conclusion, the results demonstrate that it is necessary to process features using a multi-scale CNN. The results also indicate that the utilization of the *bnf* feature alone resulted in a model accuracy of 0 for both the CT5mCGAG and G4mCGGCCGC motifs. This suggests that the *bnf* feature was ineffective in recognizing these two motifs. However, as shown in Figs. 4 and 5, available as supplementary data at *Bioinformatics* online, the *bnf* feature shows classification ability for other motifs, especially the 6 mA motifs. The results also suggest that, while the mean feature contributes the most to methylation classification, the other four features also have varying contributions.

### 3.4 Ablation experiments

We performed ablation experiments to assess the effect of the modules and features used in Nanoident. First, we individually removed each feature to assess its contribution to the accuracy of methylation typing and fine mapping. Results from the independent test set indicate that the *mean* feature contributes the most to accuracy, with a significant drop when it is removed, followed by the *std* feature. The time, *bnf*, and *ab* features contribute relatively little. Specifically, removing the *time*, *bnf*, *ab*, and basecalling features (*ab* and *bnf*) results in average accuracy decreases of 0.58%, 1.58%, 0.25%, and 1.66%, respectively. The accuracy for two motifs, CT5mCGAG and G4mCGGCCGC, increased by 4% and 3%, respectively, after removing the *bnf* feature, which is consistent with our previous experiments. The highest average accuracy is achieved when all features are integrated (Table 10, available as supplementary data at *Bioinformatics* online). We also conducted a LOOCV evaluation. The results show that removing the std feature leads to a 10.77% decrease in average model accuracy. Conversely, removing the *time*, *bnf*, *ab*, and basecalling features results in average accuracy increasing by 0.48%, 1.12%, 0.84%, and 0.78%, respectively (Table 11, available as supplementary data at *Bioinformatics* online). On the one hand, this suggests that methylation on some bacterial DNA has minimal influence on these features. On the other hand, the low precision of the basecalling model version we used introduces significant noise. With the introduction of higher-precision nanopore sequencing tools and an updated basecalling model, we expect this situation to improve significantly.

We also tested the effects of two loss functions: Focal Loss and Cross-Entropy Loss. We found that using Focal Loss improved the accuracy of Nanoident by approximately 1%. Next, we investigated the impact of the residual connections and weight block in the network architecture. The removal of the residual connections and the weight block led to a decrease of 1.1% and 0.25% in the average accuracy, demonstrating the effectiveness of these two modules (Table 12 and Fig. 6, available as supplementary data at *Bioinformatics* online). We also tested the influence of the convolution kernel size and nucleotide sequences feature on the model (Tables 13 and 14, available as supplementary data at *Bioinformatics* online).

### 3.5 De novo methylation motif enrichment

De novo motif enrichment is used to discover potential motif sequences. The motif enrichment module of nanodisco operates in two modes: manual and automatic. In manual mode, it performs well but is cumbersome to operate and heavily reliant on the personal experience of the experimenter. In automatic mode, however, its performance significantly degrades, and the program may suddenly stop or enter a dead loop during execution. For example, if all potential motifs in a cycle fail to pass the test, the original data will not be updated, and the same motifs will be found in the second round, causing the program to enter an endless loop. Nanoident addressed these issues and optimized this process by using the Mann–Whitney U test for each site-centered sequence window (length = 5). This is the first instance of using a window *P*-value to screen potential sequences. We selected 1500 sequences from single-site analysis and 500 from signal windows, combining them for MEME input. We also tried different ratios and evaluated their impact on enrichment performance (Tables 15–17, available as supplementary data at *Bioinformatics* online). Our methylation motif enrichment process is shown in Fig. 7, available as supplementary data at *Bioinformatics* online.

We tested Nanoident on three bacteria with the most motifs: *H. pylori* JP26 (20 motifs), *N. gonorrhoeae* FA 1090 (9 motifs), and *T. phaeum* DSM 12270 (8 motifs). For *H. pylori* JP26, nanodisco found 12 motifs, while Nanoident identified 16. GT6mAC (198 occurrences), GTNN6mAC (540 occurrences), and TCG6mA (562 occurrences) in *H. pylori* JP26 appeared too few times (less than an average of 4034 times) to be detected by the automatic mode of both tools. For *N. gonorrhoeae* FA 1090, nanodisco stopped after two runs and found only one motif, while Nanoident ran six times and found eight of the nine motifs. For *T. phaeum* DSM 12270, both tools found all eight motifs. Subsequently, we can conduct experiments to test whether the motifs have unique biological functions. The motif enrichment results are shown in Tables 18–20, available as supplementary data at *Bioinformatics* online. Results confirm Nanoident as a robust de novo motif enrichment tool.

## 4 Discussion

In this study, based on DNA nanopore sequencing data from different bacteria, we developed a deep-learning-based method, called Nanoident, which features a well-developed pipeline for multi-feature processing, model training and inference, and position correction post-processing for methylation typing and fine mapping in bacteria. Nanoident uses nanopore signals and basecalling features as input and applies a multi-scale neural network to process the features.

The results show that Nanoident outperforms existing methods with higher accuracy and better generalization across methylation types and species. The signal and basecalling features used in Nanoident were further analyzed and validated as effective for prediction. Additionally, we optimized the pipeline for de novo methylation motif detection, enabling the discovery of novel motifs.

In conclusion, together with nanopore sequencing, Nanoident may become a powerful method for methylation typing and fine mapping in bacteria, contributing to a better understanding and broader applications of epigenetic mechanisms in metagenomic research. In the future, we will build a larger multi-species bacterial nanopore sequencing dataset and explore downstream tasks such as metagenome methylation binning.

## Supplementary Material

btaf397_Supplementary_Data

## Data Availability

All nanopore sequencing data used in this study is publicly available at National Center for Biotechnology Information (NCBI) under the BioProject PRJNA559199.
