# Supplementary material for: Identifying DNA methylation types and methylated base positions from bacteria using nanopore sequencing with multi-scale neural network
Source: Bioinformatics. 2025 Jul 14;41(8):btaf397. doi: 10.1093/bioinformatics/btaf397 (PMC12366487; doi:10.1093/bioinformatics/btaf397)
Supplement: btaf397_Supplementary_Data [file btaf397_supplementary_data.pdf]

Supplementary: Identifying DNA methylation types and methylated base positions from bacteria using nanopore sequencing with multi-scale neural network.

Zheng Chen[1,2,3], Peng Ni[1,2,3]✉, and Jianxin Wang[1,2,3]

[1] School of Computer Science and Engineering, Central South University, Changsha, 410083, China
[2] Xiangjiang Laboratory, Changsha, 410205, China
[3] Hunan Provincial Key Lab on Bioinformatics, Central South University, Changsha, 410083, China
Correspondence to:nipeng@csu.edu.cn

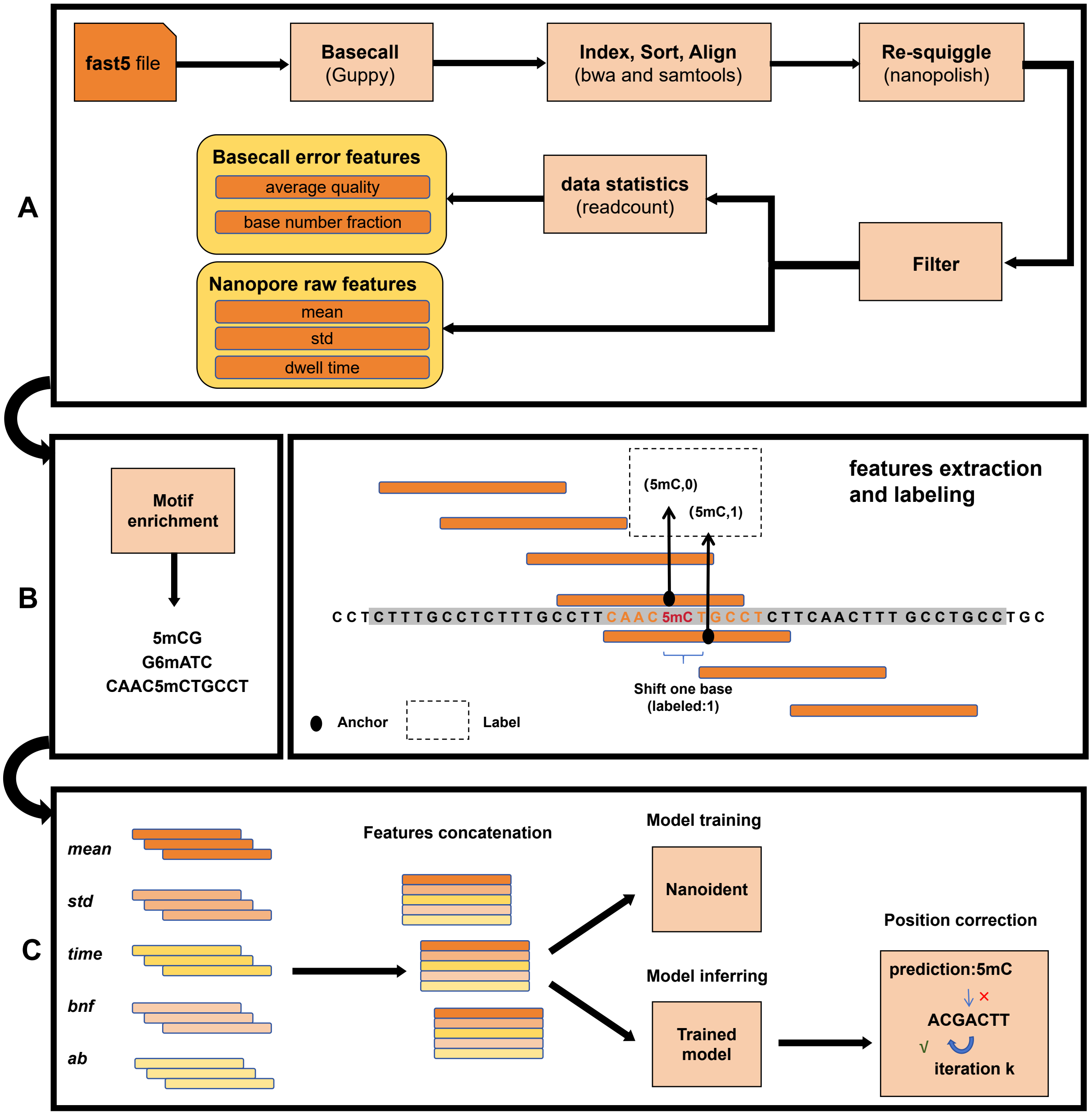

Fig. S1: A more specific pipeline of Nanoident. (A) The illustration of data preprocessing. (B) The nanopore sequencing is extracted twice to be used as a model input: The part marked in orange is motif, the part marked in grey is the first extraction, and the orange line segment represents the second extraction under different offsets. (C) Nanoident training, inferring and position correction postprocessing module.

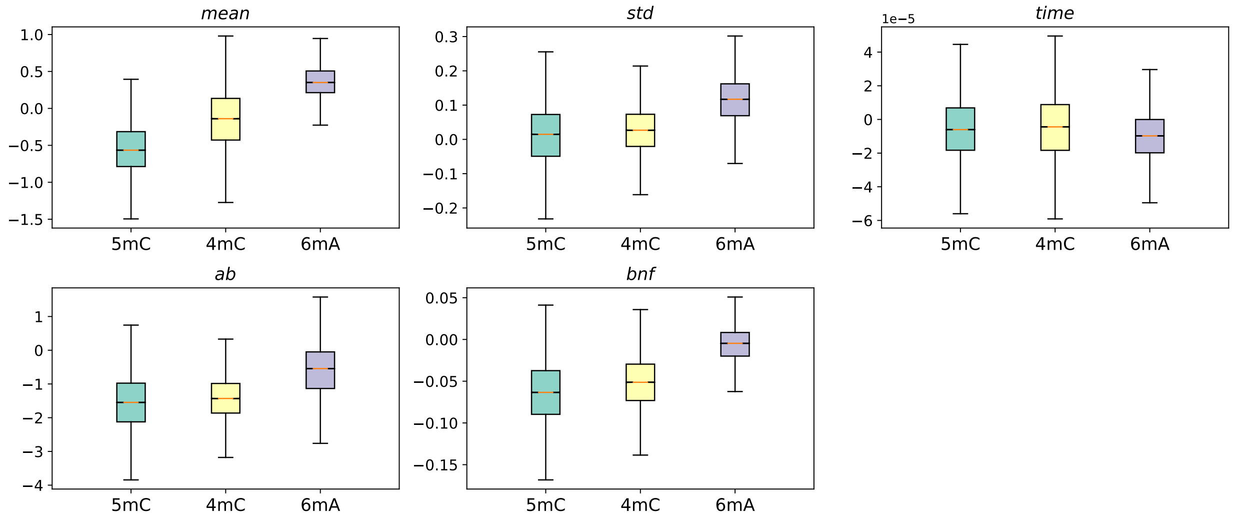

Fig. S2: Box plots of the mean values of 5 features under different methylation types.

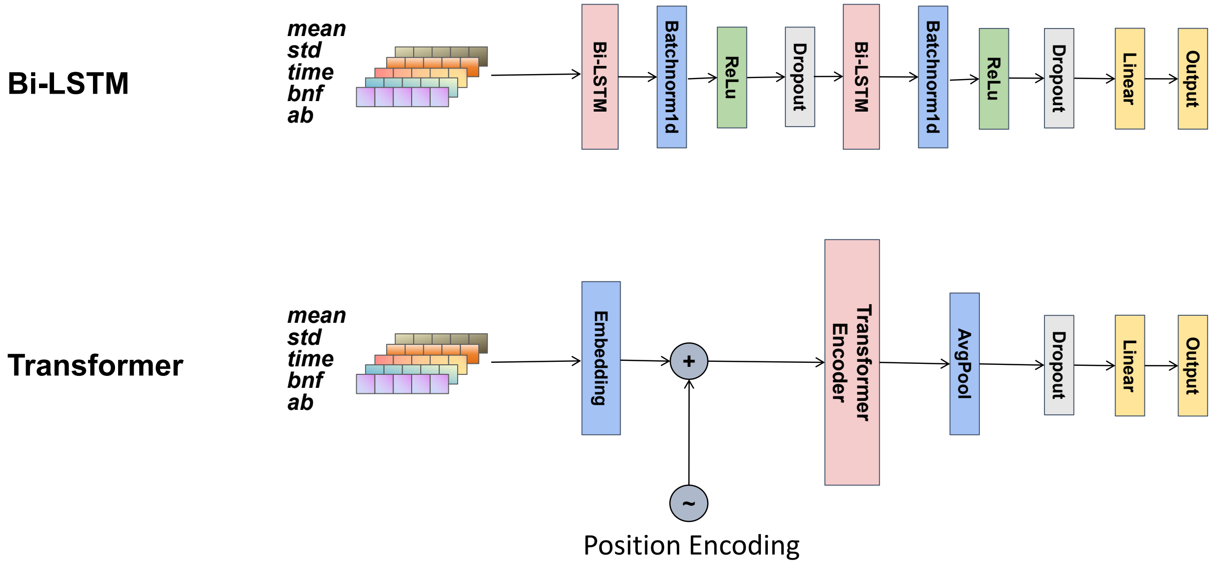

Fig. S3: Model structure built by Bi-LSTM and Transformer for identifying DNA methylation types and methylated base positions.

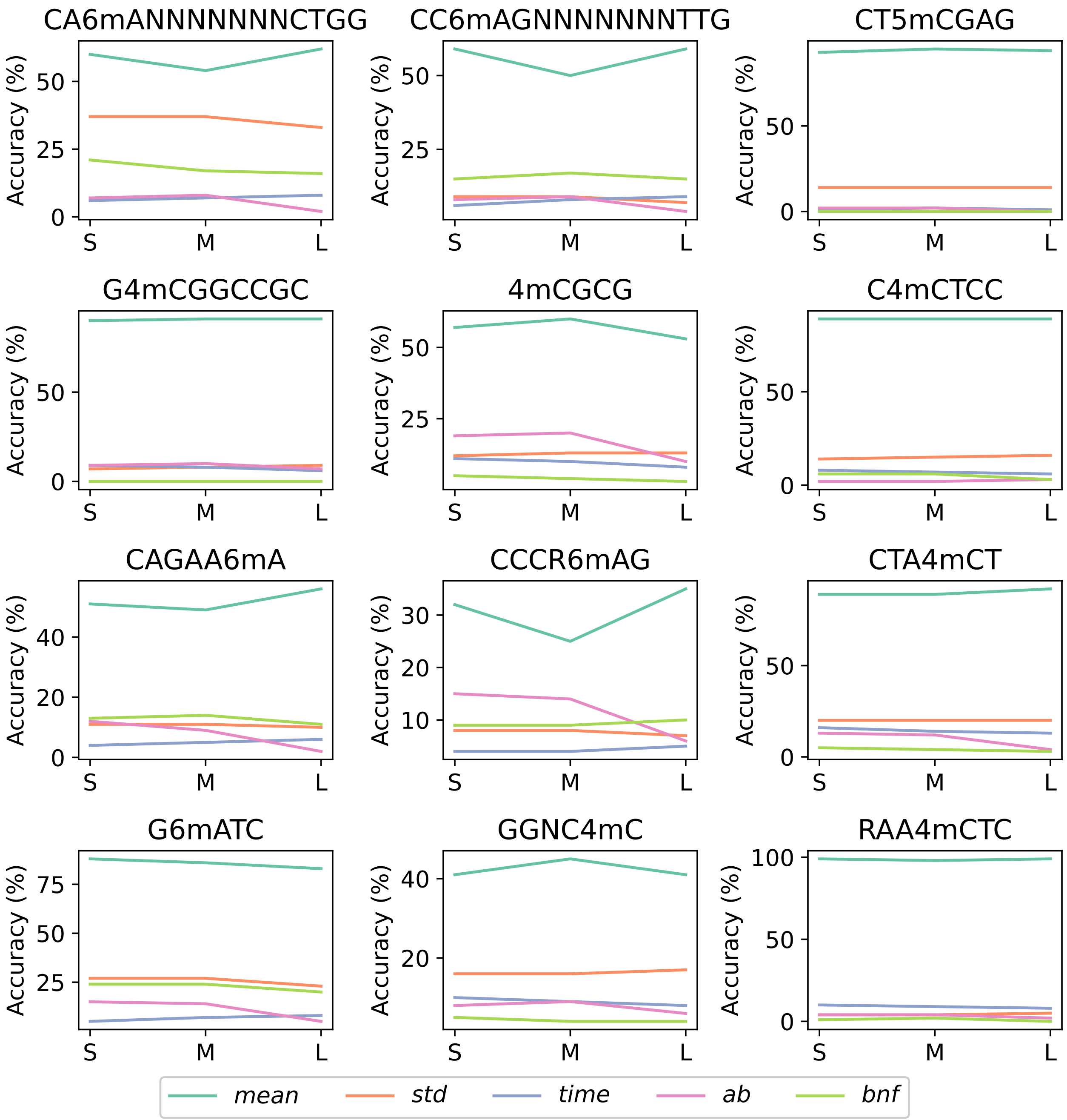

Fig. S4: The accuracy of Nanoident using five different features separately in the independent test set on three single-scales (S: small, M: medium, L: large).

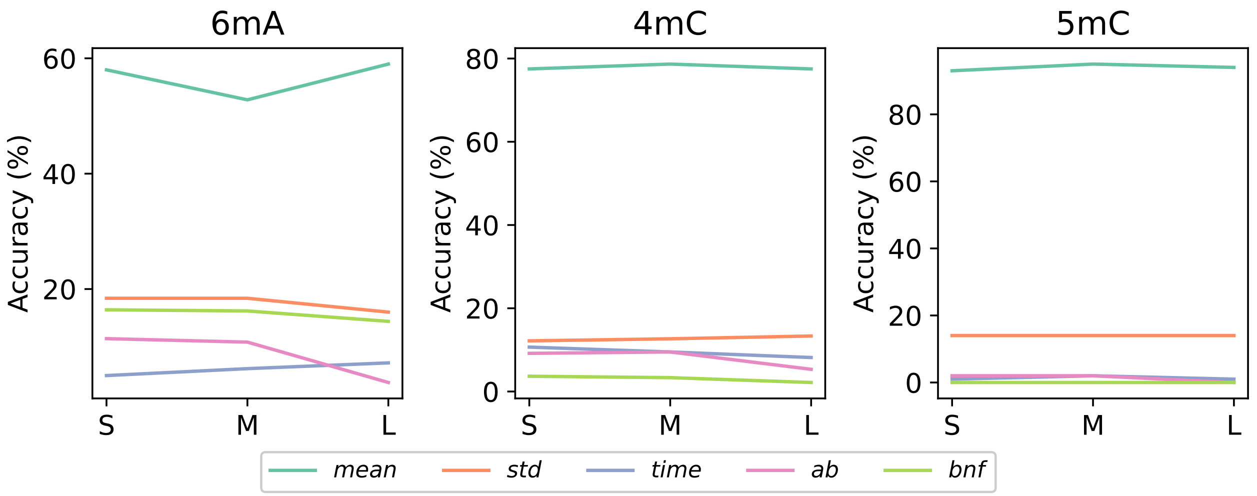

Fig. S5: The average accuracy across different methylation types of Nanoident using five different features separately in the independent test set on three single-scales (S: small, M: medium, L: large).

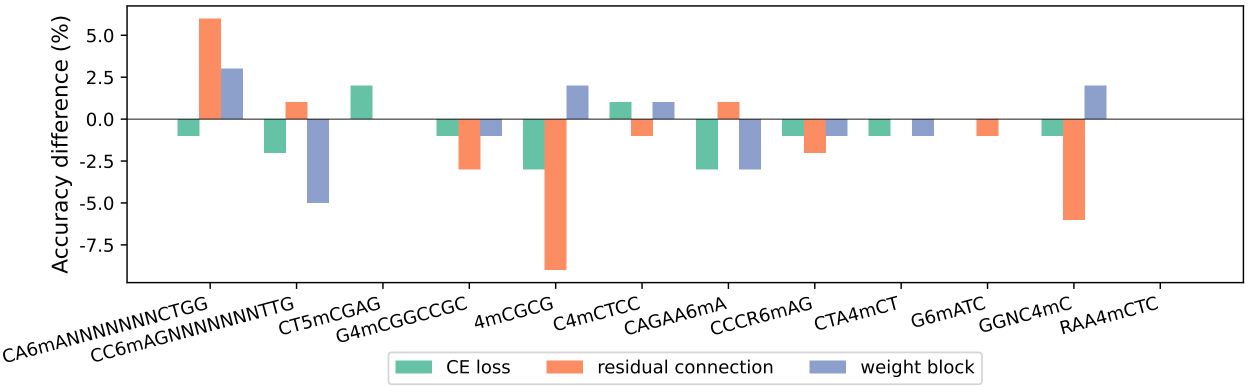

Fig. S6: The ablation experiments on the accuracy of Nanoident (The accuracy difference is defined as accuracy with that part (CE loss) - accuracy without that part (focal loss)).

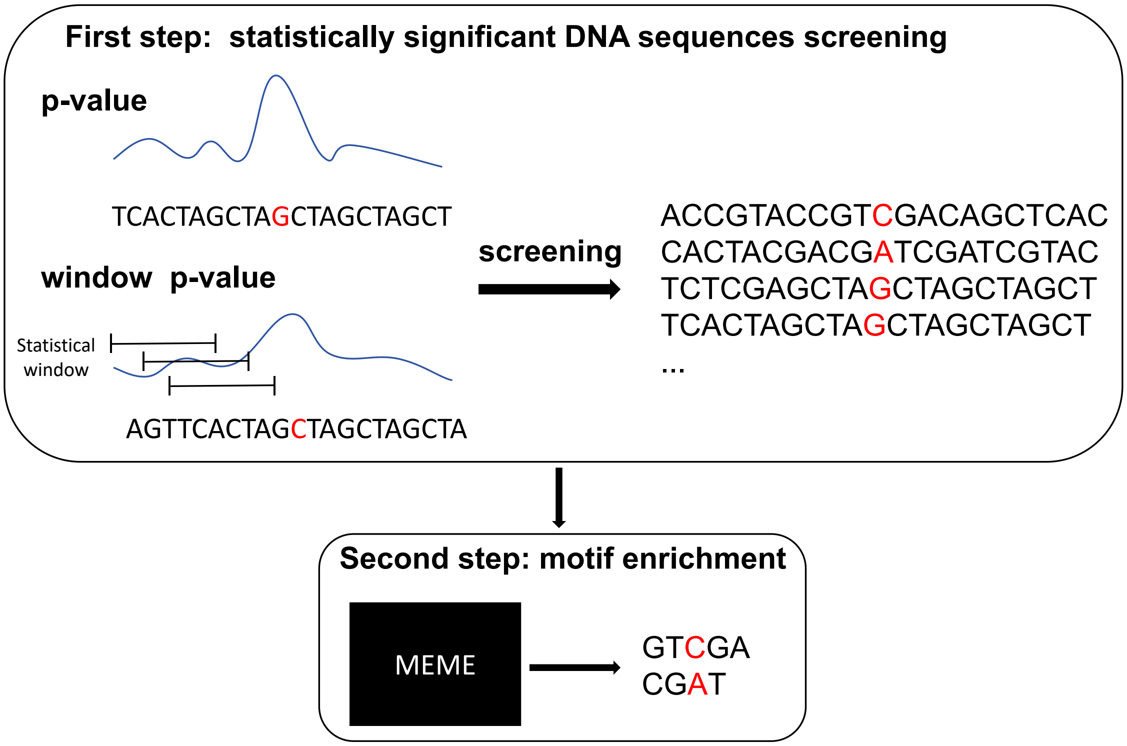

Fig. S7: motifs enrichment process.

Table S1: Training data used in this study.

Bacteria	Motif	Methylation type	Methylation position	Number occurrences
Bacillus amyloliquefaciens H	G5mCWGC	5mC	2	23726
	GGAT4mCC	4mC	5	462
Bacillus fusiformis 1226	GAT5mC	5mC	4	18428
Clostridium perfringens ATCC 13124	5mCCGG	5mC	1	780
	C6mACNNNNNRTAAA	6mA	2	279
	GAT5mC	5mC	4	8520
	GGW5mCC	5mC	4	2252
	GTAT6mAC	6mA	5	318
	TTT6mAYNNNNNGTG	6mA	4	279
	VGAC6mAT	6mA	5	2122
Escherichia coli K-12 substr. MG1655	A6mACNNNNNNGTGC	6mA	2	597
	C5mCWGG	5mC	2	24188
	G6mATC	6mA	2	38368
	GC6mACNNNNNNGTT	6mA	3	597
Helicobacter pylori JP26	4mCCGG	4mC	1	3422
	ATTA6mAT	6mA	5	876
	C6mATG	6mA	2	14318
	CRT6mANNNNNNNWC	6mA	4	1253
	CS6mAG	6mA	3	8220
	CTRY6mAG	6mA	5	1282
	CY6mANNNNNNTTC	6mA	3	1056
	G5mCGC	5mC	2	12072
	G6mAGG	6mA	2	4583
	G6mANNNNNNNTAYG	6mA	2	648
	GA6mANNNNNNTRG	6mA	3	1056
	GA6mATTC	6mA	3	298
	GG5mCC	5mC	3	2918
	GMRG6mA	6mA	5	7695
	GT6mAC	6mA	3	198
	GTNN6mAC	6mA	5	540
	T4mCTTC	4mC	2	4555
	TCG6mA	6mA	4	562
	TCNNG6mA	6mA	6	3864
	TGC6mA	6mA	4	11256
Methanospirillum hungatei JF-1	4mCTNAG	4mC	1	10908
	AG4mCT	4mC	3	11534
	CCA4mCGK	4mC	4	1396
	G6mATC	6mA	2	44388
	GCYYG6mAT	6mA	6	2024
	GTA4mC	4mC	4	15396
Neisseria gonorrhoeae FA 1090	C5mCGCGG	5mC	2	438
	G5mCCGGC	5mC	2	3174
	G6mAGNNNNNTAC	6mA	2	203
	GC6mANNNNNNNNTGC	6mA	3	1832
	GG5mCC	5mC	3	9190
	GGNN5mCC	5mC	5	3762
	GGTG6mA	6mA	5	1809
	GT6mANNNNNCTC	6mA	3	203
	RG5mCGCY	5mC	3	928

Table S2: Testing data used in this study.

Bacteria	Motif	Methylation type	Methylation position	Number occurrences
Nocardia otitidiscaviarum NEB252	CA6mANNNNNNNCTGG	6mA	3	1255
	CC6mAGNNNNNNNTTG	6mA	3	1255
	CT5mCGAG	5mC	3	6088
	G4mCGGCCGC	4mC	2	4776
Thermacetogenium phaeum DSM 12270	4mCGCG	4mC	1	12858
	C4mCTCC	4mC	2	13250
	CAGAA6mA	6mA	6	2237
	CCCR6mAG	6mA	5	2019
	CTA4mCT	4mC	4	2314
	G6mATC	6mA	2	33786
	GGNC4mC	4mC	5	19242
	RAA4mCTC	4mC	4	2975

Table S3: The average accuracy of nanodisco re-trained by us and the pre-trained model from the nanodisco paper on LOOCV evaluation and an independent dataset, respectively.

Experiment	Re-trained nanodisco	Pre-trained nanodisco
LOOCV evaluation	66.36%	66.58%
Independent dataset	73.75%	73.93%

Table S4: Comparison of accuracies between Nanoident and nanodisco on LOOCV evaluation(%).

**Bacteria**	**Motif**	**nanodisco**	**Nanoident**
Bacillus amyloliquefaciens H	G5mCWGC	56	**57**
	GGAT4mCC	40	**54**
Bacillus fusiformis 1226	GAT5mC	**95**	91
Clostridium perfringens ATCC 13124	5mCCGG	95	95
	C6mACNNNNNRTAAA	73	**76**
	GAT5mC	**95**	91
	GGW5mCC	97	97
	GTAT6mAC	**52**	43
	TTT6mAYNNNNNGTG	**66**	60
	VGAC6mAT	96	**97**
Escherichia coli K-12 substr. MG1655	A6mACNNNNNNGTGC	76	**93**
	C5mCWGG	86	**99**
	G6mATC	90	**91**
	GC6mACNNNNNNNGTT	87	**94**
Helicobacter pylori JP26	4mCCGG	66	**77**
	ATTA6mAT	90	**98**
	C6mATG	82	**96**
	CRT6mANNNNNNNWC	59	**91**
	CS6mAG	49	**81**
	CTRY6mAG	87	**93**
	CY6mANNNNNNTTC	49	**51**
	G5mCGC	62	**80**
	G6mAGG	36	**92**
	G6mANNNNNNNTAYG	81	**94**
	GA6mANNNNNNTRG	47	**76**
	GA6mATTC	78	**99**
	GG5mCC	**77**	69
	GMRG6mA	68	**93**
	GT6mAC	73	**98**
	GTNN6mAC	81	**85**
	T4mCTTC	62	**99**
	TCG6mA	75	**97**
	TCNNG6mA	74	**96**
	TGC6mA	81	**83**
Methanospirillum hungatei JF-1	4mCTNAG	44	**67**
	AG4mCT	71	**81**
	CCA4mCGK	93	**96**
	G6mATC	90	**91**
	GCYYG6mAT	95	95
	GTA4mC	87	**93**
Neisseria gonorrhoeae FA 1090	C5mCGCGG	79	**81**
	G5mCCGGC	98	**100**
	G6mAGNNNNNTAC	**50**	44
	GC6mANNNNNNNNTGC	**82**	75
	GG5mCC	**77**	69
	GGNN5mCC	95	95
	GGTG6mA	**71**	53
	GT6mANNNNNCTC	79	**94**
	RG5mCGCY	55	**71**

[1] There were 46 different motifs in 7 species of bacteria, among which GAT5mC, G6mATC and GG5mCC appeared twice in different bacteria respectively.

Table S5: Comparison of accuracies between Bi-LSTM and Transformer on LOOCV evaluation(%).

**Bacteria**	**Motif**	**Bi-LSTM**	**Transformer**
Bacillus amyloliquefaciens H	G5mCWGC	60	48
	GGAT4mCC	45	49
Bacillus fusiformis 1226	GAT5mC	83	57
Clostridium perfringens ATCC 13124	5mCCGG	95	99
	C6mACNNNNNRTAAA	75	64
	GAT5mC	89	67
	GGW5mCC	97	97
	GTAT6mAC	27	15
	TTT6mAYNNNNNGTG	60	17
	VGAC6mAT	95	97
Escherichia coli K-12 substr. MG1655	A6mACNNNNNNGTGC	76	91
	C5mCWGG	89	97
	G6mATC	93	85
	GC6mACNNNNNNGTT	91	79
Helicobacter pylori JP26	4mCCGG	67	75
	ATTA6mAT	94	98
	C6mATG	79	88
	CRT6mANNNNNNNWC	52	34
	CS6mAG	63	49
	CTRY6mAG	46	42
	CY6mANNNNNNTTC	53	35
	G5mCGC	51	10
	G6mAGG	40	77
	G6mANNNNNNNTAYG	83	80
	GA6mANNNNNNTRG	71	73
	GA6mATTC	99	100
	GG5mCC	70	68
	GMRG6mA	65	76
	GT6mAC	70	48
	GTNN6mAC	67	71
	T4mCTTC	75	98
	TCG6mA	78	77
	TCNNG6mA	80	74
	TGC6mA	56	38
Methanospirillum hungatei JF-1	4mCTNAG	66	64
	AG4mCT	69	75
	CCA4mCGK	93	95
	G6mATC	84	78
	GCYYG6mAT	91	92
	GTA4mC	92	86
Neisseria gonorrhoeae FA 1090	C5mCGCGG	72	79
	G5mCCGGC	99	100
	G6mAGNNNNNTAC	27	28
	GC6mANNNNNNNNTGC	73	57
	GG5mCC	62	56
	GGNN5mCC	93	95
	GGTG6mA	52	52
	GT6mANNNNNCTC	63	66
	RG5mCGCY	64	26

[1] There were 46 different motifs in 7 species of bacteria, among which GAT5mC, G6mATC and GG5mCC appeared twice in different bacteria respectively.

Table S6: Comparison of accuracies between Nanoident and nanodisco on two independent bacterial data(%).

**Bacteria**	**Motif**	**nanodisco**	**Nanoident**	**Nanoident +correct**	**Bi-LSTM**	**Transformer**
NO	CA6mANNNNNNNNCTGG	51	**66**	76	64	66
	CC6mAGNNNNNNNNTTG	57	**64**	89	66	65
	CT5mCGAG	**89**	85	85	76	65
	G4mCGGCCGC	**86**	82	86	78	85
TP	4mCGCG	49	**70**	74	64	63
	C4mCTCC	87	**89**	92	88	92
	CAGAA6mA	46	**55**	61	56	62
	CCCR6mAG	24	**39**	55	27	42
	CTA4mCT	86	**93**	93	91	91
	G6mATC	89	**92**	97	89	89
	GGNC4mC	38	**50**	53	46	51
	RAA4mCTC	97	**98**	98	98	98

[1] NO indicates *Nocardia otitidiscaviarum* NEB252 and TP indicates *Thermacetogenium phaeum* DSM 12270.

Table S7: Comparison of accuracies in Nanoident at different scales on LOOCV evaluation (%).

**Bacteria**	**Motif**	**S**	**M**	**L**	**Multi-Scale**
Bacillus amyloliquefaciens H	G5mCWGC	68	71	52	57
	GGAT4mCC	37	39	27	54
Bacillus fusiformis 1226	GAT5mC	93	94	86	91
Clostridium perfringens ATCC 13124	5mCCGG	92	95	96	95
	C6mACNNNNNRTAAA	77	80	60	76
	GAT5mC	92	94	91	91
	GGW5mCC	97	98	96	97
	GTAT6mAC	56	57	65	43
	TTT6mAYNNNNNGTG	79	61	65	60
	VGAC6mAT	97	96	97	97
Escherichia coli K-12 substr. MG1655	A6mACNNNNNNGTGC	96	90	89	93
	C5mCWGG	97	95	94	99
	G6mATC	91	91	85	91
	GC6mACNNNNNNGTT	95	93	94	94
Helicobacter pylori JP26	4mCCGG	80	74	69	77
	ATTA6mAT	96	96	97	98
	C6mATG	87	92	93	96
	CRT6mANNNNNNNWC	90	87	90	91
	CS6mAG	73	67	42	81
	CTRY6mAG	94	90	96	93
	CY6mANNNNNNTTC	51	55	56	51
	G5mCGC	87	76	57	80
	G6mAGG	83	81	87	92
	G6mANNNNNNNTAYG	96	94	84	94
	GA6mANNNNNNTRG	70	68	62	76
	GA6mATTC	100	100	99	99
	GG5mCC	74	57	77	69
	GMRG6mA	97	93	75	93
	GT6mAC	93	100	92	98
	GTNN6mAC	87	88	83	85
	T4mCTTC	100	97	95	99
	TCG6mA	97	95	89	97
	TCNNG6mA	96	92	86	96
	TGC6mA	85	84	79	83
Methanospirillum hungatei JF-1	4mCTNAG	63	61	56	67
	AG4mCT	70	79	79	81
	CCA4mCGK	97	96	94	96
	G6mATC	89	87	78	91
	GCYYG6mAT	96	95	93	95
	GTA4mC	89	92	91	93
Neisseria gonorrhoeae FA 1090	C5mCGCGG	86	85	87	81
	G5mCCGGC	100	99	99	100
	G6mAGNNNNNTAC	47	39	37	44
	GC6mANNNNNNNNTGC	81	73	73	75
	GG5mCC	69	57	73	69
	GGNN5mCC	95	95	93	95
	GGTG6mA	56	54	52	53
	GT6mANNNNNCTC	87	87	84	94
	RG5mCGCY	71	81	67	71

[1] There were 46 different motifs in 7 species of bacteria, among which GAT5mC, G6mATC, and GG5mCC appeared twice in different bacteria.

Table S8: Comparison of accuracies in Nanoident at different scales on two independent bacterial data (%).

**Bacteria**	**Motif**	**S**	**M**	**L**	**Multi-Scale**
Nocardia otitidiscaviarum NEB252	CA6mANNNNNNNCTGG	64	62	60	66
	CC6mAGNNNNNNNTTG	61	55	41	64
	CT5mCGAG	89	90	89	85
	G4mCGGCCGC	80	82	83	82
Thermacetogenium phaeum DSM 12270	4mCGCG	71	66	68	70
	C4mCTCC	87	86	86	89
	CAGAA6mA	50	54	56	55
	CCCR6mAG	31	38	37	39
	CTA4mCT	95	91	93	93
	G6mATC	90	91	86	92
	GGNC4mC	54	48	51	50
	RAA4mCTC	98	97	97	98

Table S9: The accuracies of the convolutional network with columns S, M, and L as input for 5 features respectively.

S						
**Bacteria**	**Motif**	***mean***	***std***	***time***	***ab***	***bnf***
Nocardia otitidiscaviarum NEB252	CA6mANNNNNNNCTGG	60	37	6	7	21
	CC6mAGNNNNNNNTTG	59	9	6	8	15
	CT5mCGAG	93	14	1	2	0
	G4mCGGCCGC	90	7	9	9	0
	mean	76	17	6	7	9
Thermacetogenium phaeum DSM 12270	4mCGCG	57	12	11	19	5
	C4mCTCC	89	14	8	2	6
	CAGAA6mA	51	11	4	12	13
	CCCR6mAG	32	8	4	15	9
	CTA4mCT	89	20	16	13	5
	G6mATC	88	27	5	15	24
	GGNC4mC	41	16	10	8	5
	RAA4mCTC	99	4	10	4	1
	mean	68	14	9	11	9
**Total**	**mean**	71	15	8	10	9

M						
**Bacteria**	**Motif**	***mean***	***std***	***time***	***ab***	***bnf***
Nocardia otitidiscaviarum NEB252	CA6mANNNNNNNCTGG	54	37	7	8	17
	CC6mAGNNNNNNNTTG	50	9	8	9	17
	CT5mCGAG	95	14	2	2	0
	G4mCGGCCGC	91	8	8	10	0
	mean	73	17	6	7	9
Thermacetogenium phaeum DSM 12270	4mCGCG	60	13	10	20	4
	C4mCTCC	89	15	7	2	6
	CAGAA6mA	49	11	5	9	14
	CCCR6mAG	25	8	4	14	9
	CTA4mCT	89	20	14	12	4
	G6mATC	86	27	7	14	24
	GGNC4mC	45	16	9	9	4
	RAA4mCTC	98	4	9	4	2
	mean	68	14	8	11	8
**Total**	**mean**	69	15	8	9	8

L						
**Bacteria**	**Motif**	***mean***	***std***	***time***	***ab***	***bnf***
Nocardia otitidiscaviarum NEB252	CA6mANNNNNNNCTGG	62	33	8	2	16
	CC6mAGNNNNNNNTTG	59	7	9	4	15
	CT5mCGAG	94	14	1	0	0
	G4mCGGCCGC	91	9	6	7	0
	mean	77	16	6	3	8
Thermacetogenium phaeum DSM 12270	4mCGCG	53	13	8	10	3
	C4mCTCC	89	16	6	3	3
	CAGAA6mA	56	10	6	2	11
	CCCR6mAG	35	7	5	6	10
	CTA4mCT	92	20	13	4	3
	G6mATC	83	23	8	5	20
	GGNC4mC	41	17	8	6	4
	RAA4mCTC	99	5	8	2	0
	mean	69	14	8	5	7
**Total**	**mean**	71	15	7	4	7

Table S10: Comparison of the impact of feature removal on the accuracy of Nanoident using the independent dataset (%).

**Bacteria**	**Motif**	**Remove *time***	**Remove *bnf***	**Remove *ab***	**Remove *std***	**Remove *mean***	**Remove base-calling features**	**all features**
NO	CA6mANNNNNNNCTGG	65	63	64	53	44	60	66
	CC6mAGNNNNNNNTTG	62	61	62	57	21	57	64
	CT5mCGAG	88	89	87	88	4	91	89
	G4mCGGCCGC	82	85	85	87	4	82	83
TP	4mCGCG	67	66	68	66	24	70	68
	C4mCTCC	89	88	90	88	28	87	86
	CAGAA6mA	55	51	53	49	36	53	56
	CCCR6mAG	37	30	35	19	30	30	37
	CTA4mCT	92	95	95	89	27	95	93
	G6mATC	92	90	91	88	50	88	86
	GGNC4mC	49	49	52	46	21	53	51
	RAA4mCTC	98	97	98	98	7	97	97

[1] NO indicates *Nocardia otitidiscaviarum* NEB252 and TP indicates *Thermacetogenium phaeum* DSM 12270.

Table S11: Comparison of the impact of feature removal on the accuracy of Nanoident by LOOCV evaluation(%).

**Bacteria**	**Motif**	***ab****	***time****	***bnf****	***std****	**(*bnf* +*ab*)***	**all**
Bacillus amyloliquefaciens H	G5mCWGC	67	63	50	47	61	57
	GGAT4mCC	53	46	56	38	56	54
Bacillus fusiformis 1226	GAT5mC	90	89	92	91	96	91
Clostridium perfringens ATCC 13124	5mCCGG	95	97	94	97	94	95
	C6mACNNNNNRTAAA	72	76	79	57	74	76
	GAT5mC	89	90	94	90	97	91
	GGW5mCC	94	96	95	97	97	97
	GTAT6mAC	56	60	59	48	65	43
	TTT6mAYNNNNNGTG	69	69	70	79	68	60
	VGAC6mAT	97	97	97	92	95	97
Escherichia coli K-12 substr. MG1655	A6mACNNNNNNGTGC	93	93	93	87	87	93
	C5mCWGG	98	98	92	98	84	99
	G6mATC	93	91	91	90	92	91
	GC6mACNNNNNNGTT	92	92	94	79	95	94
Helicobacter pylori JP26	4mCCGG	73	74	77	61	69	77
	ATTA6mAT	98	97	96	94	97	98
	C6mATG	95	94	96	90	96	96
	CRT6mANNNNNNNWC	89	89	88	59	91	91
	CS6mAG	81	76	73	66	64	81
	CTRY6mAG	97	98	95	59	96	93
	CY6mANNNNNNTTC	50	56	56	47	52	51
	G5mCGC	84	85	92	20	88	80
	G6mAGG	93	93	93	65	90	92
	G6mANNNNNNNTAYG	99	95	98	85	95	94
	GA6mANNNNNNTRG	77	75	73	66	68	76
	GA6mATTC	99	99	99	99	100	99
	GG5mCC	78	70	81	69	80	69
	GMRG6mA	98	97	95	86	94	93
	GT6mAC	98	98	98	56	98	98
	GTNN6mAC	85	86	86	81	88	85
	T4mCTTC	100	99	99	99	98	99
	TCG6mA	98	98	97	90	97	97
	TCNNG6mA	97	96	96	84	96	96
	TGC6mA	85	75	78	53	94	83
Methanospirillum hungatei JF-1	4mCTNAG	70	65	66	50	65	67
	AG4mCT	80	75	84	65	86	81
	CCA4mCGK	96	96	95	94	95	96
	G6mATC	88	90	89	89	84	91
	GCYYG6mAT	96	97	95	96	96	95
	GTA4mC	93	93	93	90	91	93
Neisseria gonorrhoeae FA 1090	C5mCGCGG	84	81	91	79	94	81
	G5mCCGGC	100	100	100	100	99	100
	G6mAGNNNNNTAC	46	48	45	28	41	44
	GC6mANNNNNNNNTGC	81	83	82	73	78	75
	GG5mCC	70	65	81	65	83	69
	GGNN5mCC	94	94	95	93	97	95
	GGTG6mA	56	55	53	48	56	53
	GT6mANNNNNCTC	91	90	88	65	87	94
	RG5mCGCY	68	82	86	48	86	71

[1] * Indicates that the feature is removed.

Table S12: Ablation experiments.

**Bacteria**	**Motif**	**CE loss**	**no residual connection**	**no weight block**
NO	CA6mANNNNNNNCTGG	65	72	69
	CC6mAGNNNNNNNTTG	62	65	59
	CT5mCGAG	87	85	85
	G4mCGGCCGC	81	79	81
	mean	74	75	74
TP	4mCGCG	67	61	72
	C4mCTCC	90	88	90
	CAGAA6mA	52	56	52
	CCCR6mAG	38	37	38
	CTA4mCT	92	93	92
	G6mATC	92	91	92
	GGNC4mC	49	44	52
	RAA4mCTC	98	98	98
	mean	72	71	73
**Total**	**mean**	73	72	73

[1] NO indicates *Nocardia otitidiscaviarum* NEB252 and TP indicates *Thermacetogenium phaeum* DSM 12270.

Table S13: The average accuracy of different convolution kernel size on the independent dataset.

**Convolution kernel size**	**Average accuracy (%)**
Large	72.75
Small	73.58

[1] we evaluated two representative configurations: Large kernel sizes (S-column: 4, 4, 4; M-column: 7, 4; L-column: 10) and small kernel sizes (S-column: 2, 2, 2; M-column: 3, 2; L-column: 4).

Table S14: The accuracy of Nanoident with and without nucleotide sequence features on the independent dataset.

**Motif**	**Accuracy with nucleotide sequence (%)**	**Accuracy without nucleotide sequence (%)**
CA6mANNNNNNNCTGG	51	66
CC6mAGNNNNNNNTTG	89	64
CT5mCGAG	31	85
G4mCGGCCGC	30	82
4mCGCG	53	70
C4mCTCC	89	89
CAGAA6mA	33	55
CCCR6mAG	56	39
CTA4mCT	100	93
G6mATC	99	92
GGNC4mC	50	50
RAA4mCTC	100	98
mean	65.08	73.58

Table S15: Comparison of motifs enrichment with different proportion parameter on *Helicobacter pylori* JP26.

**Motif**	**(1500,500)**	**(1600,400)**	**(1400,600)**
4mCCGG	RCCGG	RCCGG	-
ATTA6mAT	NATTAAT	ATTAAT	ATTAATN
C6mATG	CATG	CATG	CATG
CRT6mANNNNNNNNWC	CRTANNNNNNNWC	CRTANNNNNNNWC	CRTANNNNNNNWC
CS6mAG	CCSAG	CCSAG	-
CTRY6mAG	CTRTAG	CTRTAG	CTRYAG
CY6mANNNNNNTTC	CYANNNNNNTTC	CYANNNNNNTTC	CYANNNNNNTTC
G5mCGC	GCGC	GCGC	GCGC
G6mAGG	-	-	RNGAGG
G6mANNNNNNNTAYG	GANNNNNNNTAYG	GANNNNNNNTAYG	GANNNNNNNTAYG
GA6mANNNNNNTRG	GAANNNNNNTRG	GAANNNNNNTRG	GAANNNNNNTRG
GA6mATTC	GAATTC	GAATTC	GAATTC
GG5mCC	GGCC	GGCC	GGCC
GMRG6mA	VGMAGA/TGMGGA	NGMAGAN/NGMGGAH	GMAGAN
GT6mAC	-	-	-
GTNN6mAC	-	-	-
T4mCTTC	TCTTCNN	TCTTCWW	TCTTC
TCG6mA	-	-	-
TCNNG6mA	TCWNGA	TCWNGA	TCWNGA
TGC6mA	TGCAN	TGCANN	TGCAN

Table S16: Comparison of motifs enrichment with different proportion parameter on *Neisseria gonorrhoeae* FA 1090.

**Motif**	**(1500,500)**	**(1600,400)**	**(1400,600)**
C5mCGCGG	CCGCGG	CCGCGG	CCGCGG
G5mCCGGC	NHGCCGGC	HGCCGGCD	HGCCGGCD
G6mAGNNNNNTAC	-	-	-
GC6mANNNNNNNNTGC	GCANNNNNNNNTGC	GCANNNNNNNNTGC	GCANNNNNNNNTGC
GG5mCC	NGGCCN	NGGCCN	NGGCCN
GGNN5mCC	NGGNNCCN	NGGNNCCN	GGNNCCN
GGTG6mA	GGTGA	-	GGTGA
GT6mANNNNNCTC	GTANNNNNCTC	GTANNNNNCTC	GTANNNNNCTC
RG5mCGCY	RGCGCY	RGCGCY	NRGCGCY

Table S17: Comparison of motifs enrichment with different proportion parameter on *Thermacetogenium phaeum* DSM 12270.

**Motif**	**(1500,500)**	**(1600,400)**	**(1400,600)**
4mCGCG	NDCGCG	GRCGCG	NDCGCG
C4mCTCC	CCTCC	-	-
CAGAA6mA	CAGAAA	CAGAAA	CAGAAA
CCCR6mAG	CCCRAG	-	-
CTA4mCT	CTACT	-	-
G6mATC	NNGATC	NNGATC	NNGATC
GGNC4mC	GGNCCS	GGNCCWN	GGNCCWN
RAA4mCTC	RAACTC	RAACTC	RAACTC

Table S18: Comparison of motifs enrichment by nanodisco(auto) and Nanoident on *Helicobacter pylori* JP26.

**Motif**	**nanodisco(auto)**	**Nanoident**
4mCCGG	-	RCCGG
ATTA6mAT	ATTAAT	NATTAAT
C6mATG	CATG	CATG
CRT6mANNNNNNNNWC	CRTANNNNNNNWC	CRTANNNNNNNWC
CS6mAG	-	CCSAG
CTRY6mAG	CTRYAG	CTRTAG
CY6mANNNNNNNTTC	CYANNNNNNTTC	CYANNNNNNTTC
G5mCGC	GCGC	GCGC
G6mAGG	-	
G6mANNNNNNNTAYG	-	GANNNNNNNTAYG
GA6mANNNNNNNTRG	GAANNNNNNTRG	GAANNNNNNTRG
GA6mATTC	GAATTC	GAATTC
GG5mCC	GGCC	GGCC
GMRG6mA	-	VGMAGAN/TGMGGA
GT6mAC	-	-
GTNN6mAC	-	-
T4mCTTC	TCTTCN	TCTTCNN
TCG6mA	-	-
TCNNG6mA	TCNNGA	TCWNGA
TGC6mA	TGCAN	TGCAN

Table S19: Comparison of motifs enrichment by nanodisco(auto) and Nanoident on *Neisseria gonorrhoeae* FA 1090.

**Motif**	**nanodisco(auto)**	**Nanoident**
C5mCGCGG	-	CCGCGG
G5mCCGGC	-	NHGCCGGC
G6mAGNNNNNTAC	-	-
GC6mANNNNNNNNTGC	GCANNNNNNNNTGC	GCANNNNNNNNTGC
GG5mCC	-	NGGCCN
GGNN5mCC	-	NGGNNCCN
GGTG6mA	-	GGTGA
GT6mANNNNNCTC	-	GTANNNNNCTC
RG5mCGCY	-	RGCGCY

Table S20: Comparison of motifs enrichment by nanodisco(auto) and Nanoident on *Thermacetogenium phaeum* DSM 12270.

**Motif**	**nanodisco(auto)**	**Nanoident**
4mCGCG	NDCGCG	NDCGCG
C4mCTCC	CCTCC	CCTCC
CAGAA6mA	CAGAAA	CAGAAA
CCCR6mAG	CCCRAG	CCCRAG
CTA4mCT	CTACT	CTACT
G6mATC	NGATC	NNGATC
GGNC4mC	GGNCCS	GGNCCS
RAA4mCTC	RAACTC	RAACTC
